# DNA barcodes and ethnomedicinal use of Sharpnose guitarfish *Glaucostegus granulatus* by the locals at Keylong, Lahaul and Spiti, Himachal Pradesh

**DOI:** 10.1080/23802359.2019.1698329

**Published:** 2019-12-11

**Authors:** Ashutosh Singh, Gul Jabin, Bheem Dutt Joshi, Mukesh Thakur, Lalit Kumar Sharma, Kailash Chandra

**Affiliations:** Zoological Survey of India, Kolkata, West Bengal

**Keywords:** Guitarfish, Lahaul and Spiti, medicinal remedy, threatened marine species, wildlife trade

## Abstract

Illegal trade of fishes is common and has been in practice since ages for the support of livelihood and as dietary supplements. However, several species are protected in the Wildlife (Protection) Act, 1972 of India and their trade is restricted under CITES. In this article, we report trade of Sharpnose guitarfish (*Glaucostegus granulatus)* for the ethnomedicinal remedy, identified using DNA barcoding in the Keylong district of Lahaul and Spiti, Himachal Pradesh. This study provides the first DNA barcode of Sharpnose guitarfish. In order to handle wildlife offense cases we emphasize that a large reference database for other fishes in trade is needed.

The Guitarfishes (order Rajiformes) are cartilaginous fish belonging to the family Rhinobatidae, found mostly in tropical Indian Ocean ranging from coral reefs, offshore and mangrove habitats (Akhilesh et al. 2014). Guitarfishes have flattened boneless disk-shaped bodies, broad flat wing pectoral fins fused to the head and ventral slot-like body openings called gill slits. They share characteristics with Rays and Sawfishes (Moore 2017). They are generalist in diet and consume microalgae, clams, crabs, crustaceans and small benthic fishes (Notarbartolo di Sciara and Hillyer 1989). About 70% of the guitarfishes are globally threatened due to continuous decline and localized extinctions (Moore 2017), therefore, most of the them are listed in Convention on International Trade in Endangered Species (CITES) Appendix I & II (Hoffmann et al. 2010).

On May 29, 2018, two persons were observed selling the skin and bone of a fish (referring to them as ‘starfish’) as remedy for body pain at Keylong (Figure 1a and b; 32°31ʹ 0.42°N; 76°58ʹ 37.48°E). The seller revealed that the fish were captured in the Bay of Bengal and brought to Lahul and Spiti. The seller also mentioned his involvement in trading the fish organs that includes fins, meat, liver oil and skin in India and abroad to earn their livelihood. Fins are usually high in demand in southeast Asian countries to prepare luxury dishes (Fong and Anderson 2002) whereas gills and liver oil are used in preparation of cosmetics, medicines and dietary supplements (Rasmussen and Morrissey 2007; Averina and Kutyrev 2011).

Since the species identity could not be confirmed as there was no body remaining, a small piece of tissue collected from the specimen in the market was taken for DNA analysis. The specimen was deposited in the Center for DNA Taxonomy, Molecular Systematics Division as a sample ID F-18. Genomic DNA was extracted from the collected specimen and sequences thus obtained of 12S rRNA and cyt*b* and submitted to the GenBank with accession number MK765033 and MK570873 respectively were used in identifying the species identity. The 12S rRNA matched with highly threatened *Glaucostegus granulatus* (vulnerable) species (Figure 1c). Due to non-availability of refences database of cyt*b,* the specimen could not be identified to species level (Figure 1d).

The aquatic species play a crucial role in maintaining freshwater and marine ecosystem health by consuming individuals from mussel and sea urchin populations. If the guitarfish and related species disappear from the marine ecosystem then the population of clams, mollusks and other small invertebrates will explode because of the top down cascade effect (Jackson 2001). Hence, it is imperative to generate information on the population status of the Guitarfish as well as develop better strategies to minimize the illegal trade. We also suggest that the wildlife crime control agencies should strengthen its existing mechanism of dealing with illegal trade. DNA barcoding of fish like the Guitarfish is a logical first step in strengthening this regulation.

## Figures and Tables

**Figure 1.**
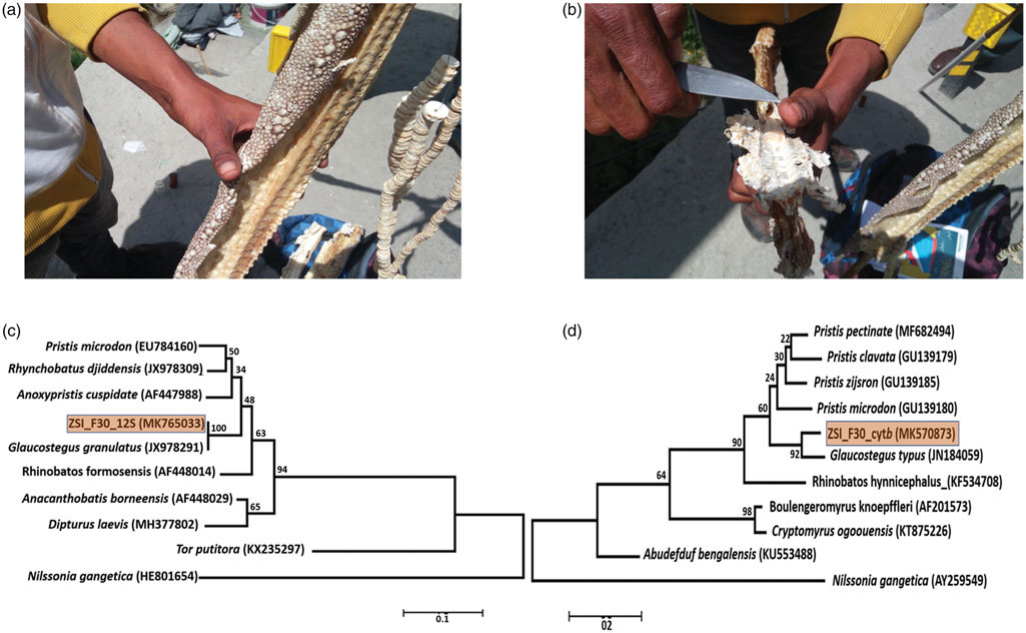
*Glaucostegus granulatus* skin (a) and bone (b). Maximum Likelihood tree based on 12S rRNA (c) and cyt *b* (d) sequence showing the relationship between the collected specimen (in box) and other suspected close relative species.
